# Associations of Nativity, Age at Migration, and Percent of Life in the U.S. with Midlife Body Mass Index and Waist Size in New York City Latinas

**DOI:** 10.3390/ijerph17072436

**Published:** 2020-04-03

**Authors:** Carmen B. Rodriguez, Ying Wei, Mary Beth Terry, Katarzyna Wyka, Shweta Athilat, Sandra S. Albrecht, Parisa Tehranifar

**Affiliations:** 1 Department of Epidemiology, Columbia University Mailman School of Public Health, New York, NY 10032, USA; cbr2121@cumc.columbia.edu (C.B.R.); mt146@cumc.columbia.edu (M.B.T.); sa3313@cumc.columbia.edu (S.A.); ssa2018@cumc.columbia.edu (S.S.A.); 2 Department of Epidemiology and Biostatistics, CUNY Graduate School of Public Health and Health Policy, New York, NY 10027, USA; Katarzyna.Wyka@sph.cuny.edu; 3 Department of Biostatistics, Columbia University Mailman School of Public Health, New York, NY 10032, USA; yw2148@cumc.columbia.edu; 4 Herbert Irving Comprehensive Cancer Center, Columbia University Medical Center, New York, NY 10032, USA

**Keywords:** migration, Caribbean Latinas, obesity

## Abstract

Migration to the U.S. has been associated with increased body size and obesity risk in Latinas, but results for Caribbean immigrant women are limited and inconclusive. Emerging evidence also suggests that early-life environment associations with women’s midlife body mass index (BMI) may be different for larger and smaller women, but this has not been tested within migration life-course history. We examined the associations of nativity and migration timing with midlife body size in a sample of majority Caribbean Latinas and whether these associations varied across the body size distribution. We used interview data from 787 self-identified Latinas (ages 40–65 years) and assessed overall obesity using BMI (kg/m^2^) and central obesity based on waist circumference (WC, cm). We used linear and quantile regression to examine the association of migration history with BMI and WC and logistic regression for the probability of obesity. Foreign birthplace, later migration age, and lower percent of life in the U.S. were associated with lower BMI and WC means and lower odds of overall and central obesity. Quantile regression showed only inverse associations in the upper quantiles of BMI and WC. For example, relative to U.S.-born women, women living <50% of their lives in the U.S. had lower BMI in the 75th BMI percentile (β = −4.10, 95% CI: −6.75, −0.81), with minimal differences in the 25th (β = 0.04, 95% CI: −1.01, 0.96) and 50th BMI percentiles (β = −1.54, 95% CI: −2.90, 0.30). Our results support that migration to and increasing time in the U.S. are associated with greater body size in midlife Latina women, with stronger influences at higher body size distribution.

## 1. Introduction

Recent evidence suggests a stabilizing trend in the prevalence of overweight and obesity in white and African American populations in the U.S., but a continued increasing prevalence in the Hispanic or Latino (hereafter Latino) population [1]. Foreign-born Latinos generally have smaller body mass index (BMI) and waist circumference and lower probability of overweight and obesity than U.S.-born Latinos, but these differences tend to diminish over successive immigrant generations [2,3]. Additionally, within the first (foreign-born) generation, these measures of body size increase with earlier age or life stage of migration and length of time spent in the U.S. [3,4,5,6,7]. The Latino population in the U.S. is highly diverse with respect to the country of origin, immigration history, ancestry, race, and cultural norms. Yet, the majority of research on migration and body size has considered the Latino population as a single group or focused on Latinos of Mexican origin [3,5,6,8,9], with only a few studies of U.S. Latinos originating from Caribbean countries (e.g., Puerto Ricans, Cubans, and Dominicans) [4,5,10]. Additionally, the majority of research has examined migration influences on the mean BMI when considering continuous measures in linear regression models or odds of obesity when considering categorical measures in logistic regression models. However, emerging recent evidence using quantile regression methods suggests that exposures in earlier life periods, such as maternal pregnancy characteristics, and birth and childhood body size and growth, can have differential associations with midlife body size for larger and smaller women [11,12,13]. This question has not been investigated in relation to nativity and migration timing but can provide additional information that extends the available findings on the association between migration and body size estimated from standard linear and logistic regression methods.

In this study, we addressed the limited empirical literature on nativity, migration timing, and body size in the understudied population of Latinas of predominantly Caribbean descent in their midlife, an important life stage when differences in body size may be particularly substantial and have significant implications for aging and chronic disease risk [14]. We also examined nativity and migration timing in relation to two different measurements of body size by considering BMI, a general measure of body mass and obesity that does not capture body composition, as well as waist circumference, which provides an assessment of central fat deposition and central obesity [15,16,17,18]. Finally, in addition to examining migration in relation to average body size and prevalence of obesity through linear and logistic regression models, respectively, we used quantile regression analysis as another method of estimation to examine the associations of migration history across different quantile levels of body size, that is, whether migration history can differentially affect smaller and larger women.

## 2. Materials and Methods 

### 2.1. Participants and Data Collection

We used data from the New York Mammographic Density (NY MaDe) Study, an ongoing study of breast cancer screening and prevention in a predominantly immigrant sample of midlife women (age range: 40–65 years). Study participants were 1069 women who were recruited during routine screening mammography appointment in Northern Manhattan from 2012 through 2018. Following the standard protocol, trained research staff conducted in-person interviews in English (41%) or Spanish (59%) to collect detailed data on sociodemographic factors, health behavior, and migration and obtained anthropometric measurements [19,20]. We limited the current analysis to 798 women who self-identified as Hispanic or Latina and further excluded participants with missing data on anthropometrics (*n* = 10) and migration history (*n* = 1), leaving a final sample of 787 women for this analysis.

All participants provided written informed consent, and the Institutional Review Board at Columbia University Medical Center approved this study (IRBAAAQ6108 and IRBAAAJ6204). 

### 2.2. Measures

#### 2.2.1. Migration History 

Based on self-reported country of birth, women were classified as U.S.-born or foreign-born to capture their nativity status. Given that Puerto Rican-born women are U.S. citizens, their migration experiences may be substantially different from those of Latina women born in other countries. We therefore created two alternative measurements of nativity that differed in whether women born in Puerto Rico (*n* = 27) were categorized as U.S.-born or foreign-born. Furthermore, since a large proportion of women in our study population were born in the Dominican Republic, we further divided foreign-born women into born in the Dominican Republic and in other countries (e.g., Puerto Rico, Cuba, Mexico). Foreign-born women reported their age at immigration to the U.S, which was used to characterize women by their life stage of migration as follows: early-life migrants (migrated when <20 years old), young adult migrants (migrated when 20–29 years old), and later adult migrants (migrated when ≥30 years old). We also used the age at migration to calculate the percent of life spent in the U.S. by subtracting the age at interview from the age at migration to the U.S., multiplied by 100 to obtain a percentage. 

#### 2.2.2. Body Size

At the time of interview, we followed a standard protocol to measure participant’s height in cm using a stadiometer, weight in pounds using a digital scale, and waist circumference in cm using a measuring tape. We converted height and weight, respectively, into meters and kilograms to calculate BMI as weight in kilograms divided by height in meters squared. Overall obesity was defined as BMI ≥ 30 kg/m^2^, and central obesity was defined as a waist circumference ≥ 88 cm based on the World Health Organization’s criteria for women [21,22].

#### 2.2.3. Sociodemographic Variables

We considered race, age, educational status, and marital status as a priori selected confounders of the association between migration and body size. Women were categorized as white Latinas and black Latinas if they only identified with either of these racial groups. Women who reported multiple racial backgrounds or chose other categories were categorized as mixed/other race Latinas. We also included data on highest educational attainment (less than high school, high school graduate, some college, bachelors, or higher degree) and marital status (married/living with partner or other).

#### 2.2.4. Statistical Analysis

We examined descriptive statistics for sociodemographic factors, nativity, and migration timing variables by the body size measures. In separate models, we examined the association of nativity and each migration timing variable with each body size measure using ordinary linear regression for continuous measures of body size and logistic regression for overall and central obesity. In addition, we used quantile regression to describe the relationship between migration history and the different quantiles of BMI and waist circumference. Unlike ordinary linear regression methods which estimate the association of the independent variable with the mean of a continuous outcome, quantile regression estimates the association across the entire distribution of a continuous outcome, using quantiles that are selected by internal percentile rank [23]. From selected potential confounders, only age and educational status were associated with both migration and body size in our data and were included in multivariable models. In secondary analyses, we repeated these multivariable models among foreign-born participants and additionally looked at age at migration as a continuous variable (presented in supplemental file). As a sensitivity analysis, we repeated our analysis of nativity status with alternative classification of Puerto Rican-born as either U.S.- or foreign-born and found similar results. We therefore limited the presentation of our results classifying Puerto Rican-born women as foreign-born. All statistical tests were 2-sided and performed using RStudio (Version 1.0.143) (RStudio, Inc, Boston, MA, USA). 

## 3. Results

About 48% and 66% of the sample, respectively, met the definition of overall and central obesity (Table 1). Prevalence of overall or central obesity increased with age (e.g., average age of 52.5 years in obese vs. 51.0 years in non-obese). Eighty-eight percent of the sample were foreign-born, of whom 87% were born in the Dominican Republic. A slightly higher percentage of foreign-born women had migrated to the U.S. during young adulthood (20–29 years, 36%) than in earlier life period (<20 years, 31%) or as older adults (≥30 years, 33%). Women who migrated during early life or during young adulthood had higher mean BMI and waist circumference than later adult migrants (mean BMI values of 31.1, 30.0, and 29.8 kg/m^2^, respectively; mean waist circumference values 94.9, 92.9, and 92.0 cm, respectively). The childhood immigrant groups also had higher prevalence of overall and central obesity than the later adult migrants (53% vs. 45% for overall obesity; 70% vs. 64% for central obesity, respectively). Similar patterns were observed by percent of life in the U.S. with women who lived <50% of their lives in the U.S. having smaller body size than those who have lived 50–99% of their lives in the U.S. (e.g., mean BMI 29.8 vs. 30.7). The average body size and prevalence of obesity also decreased with increasing educational attainment. Mixed-race Latinas had slightly higher average waist circumference than white and black Latinas. 

Table 2 shows the results from separate multivariable ordinary linear regression and logistic regression for overall and central body size measures. The results from both of these regression analyses showed lower average body size and lower odds of obesity for foreign birth, lower percent of life in the U.S, and later age at migration to the U.S., all relative to U.S.-born Latinas. Overall, body size differences between U.S.-born and foreign-born Latinas increased with less percent of life in the U.S. and later age at migration. For instance, women who immigrated to the U.S. in early life, young adulthood, and older adulthood, respectively, had 3.99 cm (95% confidence interval (CI): −6.92, −1.05), 5.99 cm (95% CI: −8.90, −3.10), and 7.20 cm (95% CI: −10.12, −4.28) smaller waist circumference and 16% (95% CI: 0.48. 1.43), 42% (95% CI: 0.34, 0.98), and 41% (95% CI: 0.34, 0.99) lower odds of central obesity, all as compared with U.S.-born Latinas. 

Results from multivariable quantile regression presented in Table 3 and Table 4 revealed overall lower BMI and waist circumference for foreign birth and later migration age, but these differences increased across higher quantiles of BMI and waist circumference, with stronger statistically significant associations with migration history limited to the upper quantiles (≥75th percentile) of BMI and waist circumference. For instance, compared with U.S.-born, foreign-born Latinas who spent less than 50% of their lives in the U.S. had 1.50 to 3.84 cm smaller average waist circumference in the 10th through the 50th percentiles of waist circumference, while 12.62 cm (95% CI: −17.96, −4.88) and 14.47 cm (95% CI: −23.33, −8.70) smaller waist circumference were observed for the same migration comparison in the 75th and 90th percentiles, respectively (Table 4). Foreign-born Latinas also had smaller waist circumference than the U.S.-born Latinas (e.g., β = −11.55, 95% CI: −16.68, −4.15 and β = −14.76, 95% CI: −21.28, −7.26, when comparing women born in the Dominican Republic with women born in the U.S. for the 75th and 90th percentiles, respectively). We observed similar patterns of inverse associations for upper quantiles of BMI and WC for these same models when we restricted the sample to foreign-born women and also when we modeled age at migration as a continuous variable in foreign-born women (Figure S1, Tables S1 and S2). 

## 4. Discussion

The rationale for undertaking this study was to further elucidate the associations of nativity and migration timing with body size in midlife Latinas, expanding the available literature in two specific ways. First, we were motivated by the proposition that the heterogeneity of the Latino population is important in health research but is not adequately examined in research on obesity. Importantly, as non-Mexican ethnic groups are frequently underrepresented in national studies of the Latino population [24,25], most research conclusions may not be applicable to other Latino subgroups. In this cross-sectional analysis, we examined the impact of migration history on body size using a sample mostly comprised of women born in the Dominican Republic and other Caribbean countries that represent a growing population in the U.S., with an 85% increase in size between 2000 and 2010 [26] (an increase higher than that of any other subgroup with a female-dominated migratory flow [27]). Second, we extended our analysis to compare the associations of migration timing with both overall body size and central body fat distribution, using several methods to understand the risk of obesity as well as the distribution of body size and composition associated with migration timing. 

Our overall findings support that foreign-born Latina women with lower proportion of life in the U.S. and older age at migration to the U.S. have smaller average BMI and waist circumference and lower odds of both overall and central obesity compared to U.S.-born Latina women. We also report that migration variables are associated with BMI and waist circumference but that these associations differ for larger (higher quantiles of body size distribution) and smaller (lower quantiles of body size distribution) women. Specifically, we found strong statistically significant inverse associations between migration variables and BMI and WC in the upper quantiles (≥75th percentile) of BMI and WC, suggesting that standard regression methods may not fully describe the differential association of nativity and migration timing across the body size distributions. 

Our results are broadly consistent with the evidence from prior studies conducted in Latino populations, showing lower body size in the foreign-born than in the U.S.-born Latinos [2,3,4,5,6,7,8,10,21,24,28,29,30,31]. Fewer studies have disaggregated Latino population by heritage or country of origin and have yielded inconclusive results for migration and body size for individuals born in or born to parents from Caribbean countries including Puerto Rico, Cuba, or the Dominican Republic [5,8,10,28]. In agreement with our results, The Hispanic Community Health Study/Study of Latinos (SOL) showed associations between length of residence in the U.S. and obesity in all Hispanic/Latino subgroups defined by their birthplace. Additionally, a study using the National Health Interview Survey (NHIS) examining trends in U.S. obesity among 30 immigrant groups found lower prevalence of obesity for foreign-born Cubans, particularly more recent immigrants (<15 years in the U.S.), than for U.S.-born Non-Hispanic whites, for whom the prevalence of obesity was similar to that for U.S.-born Cubans. However, in the same study, the prevalence of obesity in recent Puerto Rican immigrants was similar to that in U.S.-born Non-Hispanic whites, while the prevalence in U.S.-born Puerto Rican and long-term Puerto Rican immigrants was even higher than in U.S.-born Non-Hispanic whites [8]. In our study, we were able to consider women born in the Dominican Republic as a single group, allowing us to contribute new data that suggest increasing overall and central obesity prevalence and body size as compared to U.S.-born Latinas, the majority of whom had at least one Caribbean-born parent. Together, these limited but intriguing available findings underscore the possibility of heterogeneity of associations between migration timing and body size that may exist even among Caribbean Latina subgroups. 

The most common explanation for increasing body size with time in the U.S. involves the adaptation to prevalent lifestyles and environments in the U.S. that promote weight gain and/or abandonment of traditional behaviors that promote healthy weight (e.g., having food routines); these processes are likely to increase with later age at migration to and longer residence in the U.S. [32,33]. In studies that have adjusted for lifestyle factors such as diet and physical activities, migration timing associations with obesity and body size persist [3,6,8]. Issues inherent in the migration process such as changes in employment, household dynamics, and social support systems have also been proposed as a possible explanation for body size changes, but require additional empirical research [32,33]. Understanding the mechanisms underlying the immigration–obesity association can provide important insight for efforts to prevent weight gain in immigrants as they settle into their host countries. Ultimately, unpacking these mechanisms requires carefully designed migrant studies with prospective data to capture changes in immigrants’ environment, behaviors, and body size. 

Our results are limited by the cross-sectional design of our study which allowed only for examination of prevalence of obesity. We were unable to consider the effect of generational status and linguistic acculturation on body size, given that the vast majority of our study population was first-generation (foreign-born) and monolingual Spanish speaking. Larger and more diverse study populations are also needed to disaggregate the U.S.-born group (e.g., U.S.-born women born to Dominican-born parent) as well as capture the heterogeneity that may exist in foreign-born groups. With the exception of Dominican Republic-born women, we were unable to disaggregate other groups by country of origin due to the small number of women born in other countries. The majority of studies of body size changes in Latino immigrants have been conducted in the U.S., limiting our current understanding of whether there is a general process of increases in BMI and waist circumference that accompanies immigration or whether these patterns are specific to the host countries [5].

One of the main strengths of our study is examining the differences in the associations between migration and body size across the entire distribution of body size through the use of quantile regression, as prior research considering other exposures have shown differential associations across the distribution of midlife BMI. As shown here, the magnitude of the associations varied by quantiles of BMI and waist circumference, suggesting that ordinary linear regression overestimated the associations in the lower quantiles and underestimated the associations in the upper quantiles. We considered other methods, such as logistic regression and relative risk regression (results not shown), as these are flexible and easy to interpret; however, these required a priori choice of a cutoff values. With quantile regression, we were able to select quantiles based on the internal percentile rank, rather than using absolute cutoff values. While we found similar overall results across these multiple statistical approaches, we were able to provide new data to demonstrate that migration may affect women’s body size differently depending on where women lie in the distribution of BMI and waist circumference. 

## 5. Conclusions

Migration timing captured through foreign birthplace, later age at migration, and less time lived in the U.S. is associated with lower BMI and waist circumference as well as with lower probability of overall and central obesity in middle-aged Latina women from Caribbean heritage, with stronger associations observed at the higher distribution of BMI and waist circumference. 

## Figures and Tables

**Table 1:** Characteristics of Latina women in the New York Mammographic Density Study (*n* = 787).

Characteristics	Body Mass Index			Waist Circumference		
	Mean (SD)	Median (IQR)	% Overall Obese	Mean (SD)	Median (IQR)	% Central Obese
**Educational Attainment**						
Less than High School (242)	31.3 (5.9)	30.9 (7.1)	132 (54.5)	94.8 (12.1)	94.0 (16.0)	171 (70.7)
High School Graduate (184)	30.5 (5.3)	29.6 (6.0)	87 (47.3)	93.8 (12.3)	93.0 (15.0)	120 (65.2)
Some College (179)	30.1 (5.4)	29.1 (7.0)	76 (42.5)	93.2 (11.4)	91.0 (15.5)	115 (64.2)
Bachelor’s or higher degree (182)	29.6 (5.9)	28.9 (7.4)	81 (44.5)	92.9 (13.0)	91.0 (16.8)	113 (62.1)
**Marital Status**						
Single, never married (159)	30.8 (5.9)	30.1 (7.9)	83 (52.2)	95.9 (12.9)	94.0 (18.3)	114 (71.7)
Married or currently living with a partner (334)	30.2 (5.2)	29.8 (6.9)	161 (48.2)	93.0 (11.5)	92.3 (14.0)	215 (64.4)
Other (292)	30.5 (6.0)	32.1 (1.7)	130 (44.5)	93.4 (12.5)	91.0 (16.5)	188 (64.4)
**Race**						
White (210)	30.7 (6.0)	30.1 (8.1)	106 (50.5)	93.6 (13.0)	91.5 (17.8)	126 (60.0)
Black (172)	30.5 (5.7)	29.7 (6.9)	83 (48.3)	92.5 (12.1)	91.0 (16.0)	107 (62.2)
Mixed race or other (405)	30.3 (5.5)	29.4 (6.9)	187 (46.2)	94.4 (11.8)	93.5 (15.0)	286 (70.6)
**Nativity**						
U.S.-born (98)	31.4 (7.3)	30.3 (10.4)	50 (51.0)	97.4 (16.1)	94.0 (25.1)	68 (69.4)
Foreign-Born Dominican Republic (597)	30.3 (5.1)	29.6 (6.6)	286 (47.9)	92.9 (11.1)	92.0 (15.0)	393 (65.8)
Foreign-born Other (92)	30.6 (7.1)	29.3 (8.4)	40 (43.5)	95.3 (13.5)	93.0 (16.9)	58 (63.0)
**Among Foreign-Born**						
**% of Life Spent in the U.S.**						
50%–99% (389)	30.7 (5.7)	29.9 (7.40)	193 (49.6)	94.1 (11.87)	93 (16.0)	261 (67.1)
<50% (300)	29.8 (4.9)	29.4 (6.36)	133 (44.3)	92.1 (10.81)	91 (14.3)	190 (63.3)
**Age at Migration to the U.S.**						
Migrated when <20 years old (early life, 216)	31.1 (5.8)	30.3 (7.3)	115 (53.2)	94.9 (11.9)	93.8 (16.0)	152 (70.4)
Migrated when 20–29 years old (young adult, 249)	30.0 (5.3)	28.9 (6.8)	111 (44.6)	92.9 (11.7)	91.5 (15.0)	156 (62.7)
Migrated when ≥30 years old (later adult, 224)	29.8 (5.0)	29.3 (6.5)	100 (44.6)	92.0 (10.6)	91.0 (15.0)	143 (63.8)

**Table 2:** Multivariable associations of nativity (Panel A), percent of life spent in the U.S. (Panel B), and age at migration (Panel C) with body mass index and waist circumference using linear and logistic regressions.

**Panel**	**Migration**	**Body Mass Index**		**Waist Circumference**	
		**Linear Model ^ǂ^**	**Logistic Regression ^¥^**	**Linear Model ^ǂ^**	**Logistic Regression ^¥^**
		**β (95% CI)**	**OR (95% CI)**	**β (95% CI)**	**OR (95% CI)**
**Panel A**	**Nativity**				
	U.S.-born	Ref	Ref	Ref	Ref
	Foreign-Born Dominican Republic	−2.0 (−3.24, −0.76)	0.68 (0.43, 1.07)	−6.10 (−8.76, −3.44)	0.67 (0.40, 1.07)
	Foreign-born Other	−1.57 (−3.18, 0.04)	0.57 (0.31, 1.02)	−3.78 (−7.24, −0.31)	0.58 (0.31, 1.09)
**Panel B**	**% of Life Spent in the U.S.**				
	U.S.-born	Ref	Ref	Ref	Ref
	50%–99%	−1.64 (−2.94, −0.35)	0.71 (0.44, 1.14)	−4.97 (−7.75, −2.20)	0.69 (0.41, 1.14)
	<50%	−2.24 (−3.54, −0.94)	0.62 (0.38, 0.99)	−6.65 (−9.37, −3.80)	0.62 (0.37, 1.02)
**Panel C**	**Age at Migration to the U.S.**				
	U.S.-born	Ref	Ref	Ref	Ref
	Migrated when <20 years old	−1.10 (−2.46, 0.27)	0.86 (0.52, 1.41)	−3.99 (−6.92, −1.05)	0.84 (0.48, 1.43)
	Migrated when 20–29 years old	−2.24 (−3.58, −0.89)	0.60 (0.36, 0.98)	−5.99 (−8.90, −3.10)	0.58 (0.34, 0.98)
	Migrated when ≥30 years old	−2.43 (−3.79, −1.07)	0.58 (0.35, 0.95)	−7.20 (−10.21, −4.28)	0.59 (0.34, 0.99)

All models were adjusted for age and educational attainment. ^ǂ^ Parameter estimates and 95% Confidence Intervals (CI) from ordinary least-squares regression. ^¥^ Odds ratio (OR) and 95% Confidence Intervals (CI) from logistic regression model comparing waist circumference ≥ 88 cm (central obesity) with waist circumference < 88 cm and body mass index ≥ 30 kg/m^2^ (overall obesity) with body mass index < 30 kg/m^2^.

**Table 3:** Multivariable associations of nativity (Panel A), percent of life spent in the U.S. (Panel B), and age at migration (Panel C) with body mass index using quantile regression.

Panel	Migration	Quantile ^ᵻ^				
		10th Percentile	25th Percentile	50th Percentile	75th Percentile	90th Percentile
		β (95% CI)	β (95% CI)	β (95% CI)	β (95% CI)	β (95% CI)
**Panel A**	**Nativity**					
	U.S.-born	Ref	Ref	Ref	Ref	Ref
	Foreign-Born Dominican Republic	0.23 (−1.25, 2.93)	0.08 (−0.94, 1.14)	−1.30 (−2.80, 0.47)	−3.72 (−6.41, −0.43)	−6.26 (−9.97, −4.52)
	Foreign-born Other	0.41 (−2.43, 2.90)	−0.90 (−2.22, 0.65)	−1.96 (−3.12, 0.27)	−2.62 (−6.70, 0.63)	−4.25 (−9.14, 0.95)
**Panel B**	**% of Life Spent in the U.S.**					
	U.S.-born	Ref	Ref	Ref	Ref	Ref
	50–99%	0.23 (−1.21, 2.74)	−0.15 (−1.33, 1.04)	−1.54 (−2.80, 0.60)	−2.86 (−5.38, 0.80)	−5.58 (−8.98, −3.22)
	<50%	0.03 (−1.29, 2.76)	0.04 (−1.01, 0.96)	−1.54 (−2.90, 0.30)	−4.10 (−6.75, −0.81)	−7.32 (−10.81, −4.90)
**Panel C**	**Age at Migration to the U.S.**					
	U.S.-born	Ref	Ref	Ref	Ref	Ref
	Migrated when <20 years old	0.90 (−0.96, 2.82)	0.60 (−0.68, 1.95)	−0.46 (−2.18, 1.55)	−2.21 (−5.0, 1.16)	−4.82 (−8.68, −.46)
	Migrated when 20–29 years old	0.11 (−1.38, 2.30)	−0.36 (−1.56, 0.69)	−1.55 (−3.16, 0.32)	−3.88 (−6.61, −0.46)	−5.98 (−9.93, −3.59)
	Migrated when ≥30 years old	−0.04 (−1.51, 1.93)	0.04 (−0.87, 1.08)	−1.71 (−3.12, 0.35)	−4.34 (−7.07, −0.88)	−7.41 (−10.54, −4.90)

^ᵻ^ Age- and education-adjusted parameter estimates and 95% Confidence Intervals (CI) based on quantile regression methods, by quantile.

**Table 4:** Multivariable associations of nativity (Panel A), percent of life spent in the U.S. (Panel B), and age at migration (Panel C) with waist circumference using quantile regression.

Panel	Migration	Quantile ^ᵻ^				
		10th Percentile	25th Percentile	50th Percentile	75th Percentile	90th Percentile
		β (95% CI)	β (95% CI)	β (95% CI)	β (95% CI)	β (95% CI)
**Panel A**	**Nativity**					
	U.S.-born	Ref	Ref	Ref	Ref	Ref
	Foreign-Born Dominican Republic	−1.43 (−5.05, 3.65)	−2.08 (−4.27, 0.71)	−3.24 (−8.28, 2.15)	−11.55 (−16.68, −4.15)	−14.76 (−21.28, −7.26)
	Foreign-born Other	0.63 (−4.10, 5.03)	−1.96 (−4.45, 1.56)	−2.59 (−7.0, 2.68)	−8.50 (−16.80, 0.86)	−8.40 (−13.95, 1.49)
**Panel B**	**% of Life Spent in the U.S.**					
	U.S.-born	Ref	Ref	Ref	Ref	Ref
	50–99%	−1.05 (−4.94, 4.06)	−1.87 (−3.69, 1.15)	−2.98 (−8.0, 2.78)	−10.40 (−16.24, −2.34)	−10.71 (−18.93, −4.92)
	<50%	−1.50 (−4.67, 3.82)	−2.31 (−4.49, 0.66)	−3.84 (−10.04, 1.49)	−12.62 (−17.96, −4.88)	−14.47 (−23.23, −8.70)
**Panel C**	**Age at Migration to the U.S.**					
	U.S.-born	Ref	Ref	Ref	Ref	Ref
	Migrated when <20 years old	−0.89 (−4.02, 3.89)	−1.68 (−3.56, 2.49)	−2.20 (−6.61, 2.18)	−9.26 (−14.75, −0.60)	−9.51 (−18.15, −3.98)
	Migrated when 20–29 years old	−0.88 (−5.34, 2.70)	−1.96 (−4.39, 0.97)	−3.98 (−9.40, 0.71)	−11.80 (−17.96, −.34)	−13.60 (−22.43, −7.09)
	Migrated when ≥30 years old	−2.32 (−6.11, 2.37)	−2.67 (−4.56, 0.77)	−4.73 (−10.30, 0.49)	−13.10 (−18.60, −4.73)	−16.18 (−24.90, −9.27)

^ᵻ^ Age- and education-adjusted parameter estimates and 95% Confidence Intervals (CI) based on quantile regression methods, by quantile.

## Supplementary Materials

The following are available online at https://www.mdpi.com/1660-4601/17/7/2436/s1, Figure S1: Age and education adjusted quantile regression for the associations of age at migration and body size among foreign- born participants, Table S1: Multivariable associations of nativity (Panel A), percent life spent in the U.S. (Panel B) and age at migration (Panel C) with body mass index using quantile regression among foreign born women (*n* = 689)., Table S2: Multivariable associations of nativity (Panel A), percent life spent in the U.S. (Panel B) and age at migration (Panel C) with waist circumference using quantile regression among foreign born women (*n* = 689).
