# Supplementary material for: Associations of Nativity, Age at Migration, and Percent of Life in the U.S. with Midlife Body Mass Index and Waist Size in New York City Latinas
Source: Int J Environ Res Public Health. 2020 Apr 3;17(7):2436. doi: 10.3390/ijerph17072436 (PMC7178279; doi:10.3390/ijerph17072436)
Supplement: Supplementary file 1 [file ijerph-17-02436-s001.pdf]

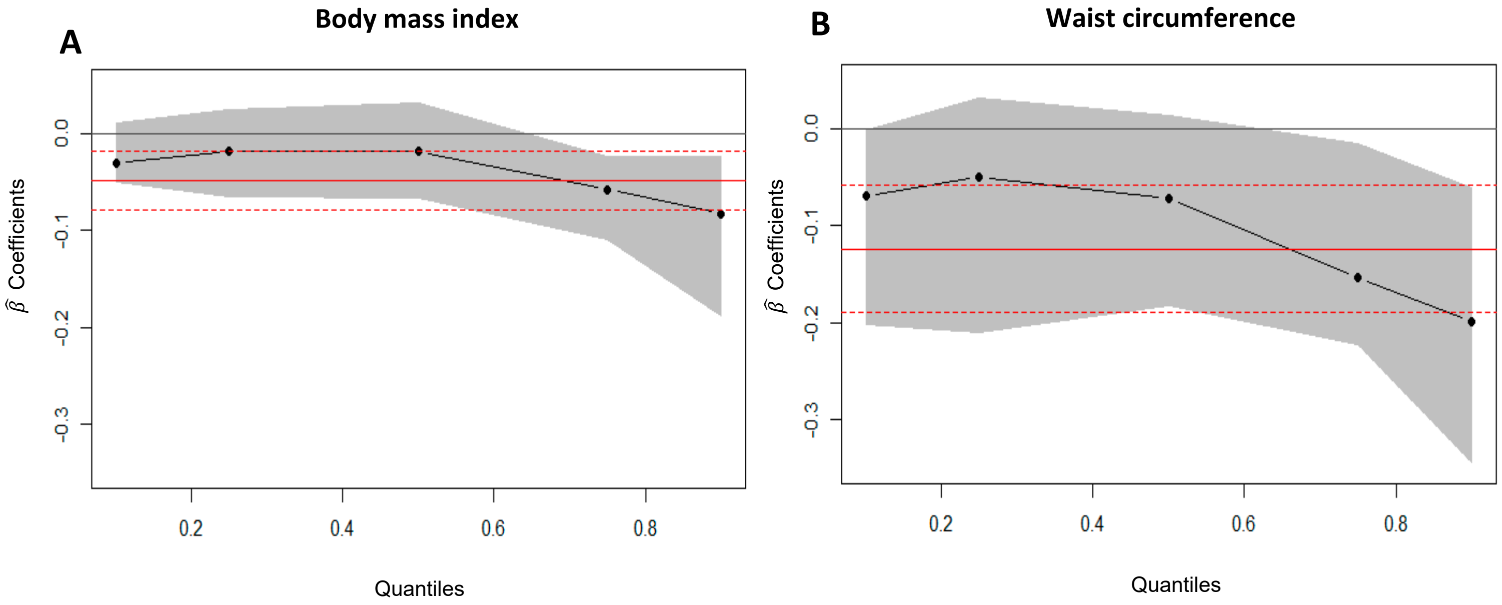

Figure S1. Age and education adjusted quantile regression for the associations of age at migration and body size among foreign- born participants. The x-axis represents the quantile level (e.g., 0.2 = 20th percentile) of body size, and the y-axis represents the corresponding quantile-specific parameter estimates. The oval shapes show β estimates for quantiles 0.10, 0.25, 0.50, 0.75 and 0.90, shaded polygon shows 95% confidence intervals from quantile; solid line (red) shows results from OLS regression and dashed lines are corresponding 95% confidence intervals.

Table S1. Multivariable associations of nativity (Panel A), percent life spent in the U.S. (Panel B) and age at migration (Panel C) with body mass index using quantile regression among foreign born women (*n* = 689).

		Quantileᵻ				
		**10th percentile**	**25th percentile**	**50th percentile**	**75th percentile**	**90th percentile**
		β (95% CI)	β (95% CI)	β (95% CI)	β (95% CI)	β (95% CI)
	**Nativity**					
**Panel A**	Foreign Born Dominican Republic	-0.18 (-0.82, 2.76)	1.21 (-0.21, 2.03)	0.69 (-1.13, 1.82)	-1.01 (-2.80, 1.52)	-1.98 (-9.12, 1.58)
	Foreign born Other	Ref	Ref	Ref	Ref	Ref
	**% of Life spent in the U.S.**					
**Panel B**	50-99%	Ref	Ref	Ref	Ref	Ref
	< 50%	-0.38 (-0.90, 0.84)	0.06 (-0.81, 0.86)	-0.18 (-1.21, 0.93)	-1.38 (-2.50, -0.34)	-1.74 (-3.65, 0.55)
	**Age at Migration to the U.S.**					
**Panel C**	Migrated < 20 years old	Ref	Ref	Ref	Ref	Ref
	Migrated 20-29 years old	-0.77 (-1.47, 0.60)	-0.88 (-2.10, -0.07)	-1.07 (-2.51, 0.01)	-1.76 (-2.73, -0.48)	-1.13 (-3.75, 0.87)
	Migrated ≥ 30 years old	-0.98 (-1.78, 0.30)	-0.55 (-1.67, 0.30)	-1.11 (-2.34, 0.02)	-2.09 (-3.17, -0.91)	-2.65 (-4.95, 0.41)

All models are adjusted for age and educational attainment. ᵻ Parameter estimates and 95% Confidence Intervals (CI) based on quantile regression methods, by quantile.

Table S2. Multivariable associations of nativity (Panel A), percent life spent in the U.S. (Panel B) and age at migration (Panel C) with waist circumference using quantile regression among foreign born women (*n* = 689).

		Quantileᵻ				
		**10th percentile**	**25th percentile**	**50th percentile**	**75th percentile**	**90th percentile**
		β (95% CI)	β (95% CI)	β (95% CI)	β (95% CI)	β (95% CI)
	**Nativity**					
**Panel A**	Foreign Born Dominican Republic	-1.64 (-3.56, 1.07)	-0.58 (-2.63, 2.15)	0.16 (-4.0, 2.95)	-1.77 (-10.61, 1.89)	-5.88 (-14.41, -2.86)
	Foreign born Other	Ref	Ref	Ref	Ref	Ref
	**% of Life spent in the U.S.**					
**Panel B**	50-99%	Ref	Ref	Ref	Ref	Ref
	< 50%	-0.58 (-3.45, 1.24)	-0.55 (-1.91, 1.51)	-1.24 (-3.37, 0.81)	-2.25 (-4.92, 0.49)	-3.70 (-6.54, -0.15)
	**Age at Migration to the U.S.**					
**Panel C**	Migrated < 20 years old	Ref	Ref	Ref	Ref	Ref
	Migrated 20-29 years old	-0.73 (-2.90, 1.09)	-0.74 (-3.88, 0.72)	-1.88 (-4.41, 0.12)	-2.52 (-5.93, 0.80)	-3.61 (-7.44, -0.49)
	Migrated ≥ 30 years old	-1.59 (-4.69, 0.34)	-1.44 (-3.53, 1.12)	-2.31 (-5.44, 0.43)	-3.50 (-7.52, -1.04)	-5.69 (-10.95, -2.84)

All models are adjusted for age and educational attainment. ᵻ Parameter estimates and 95% Confidence Intervals (CI) based on quantile regression methods, by quantile.
